# PISCOeo_pm, a reference evapotranspiration gridded database based on FAO Penman-Monteith in Peru

**DOI:** 10.1038/s41597-022-01373-8

**Published:** 2022-06-17

**Authors:** Adrian Huerta, Vivien Bonnesoeur, José Cuadros-Adriazola, Leonardo Gutierrez, Boris F. Ochoa-Tocachi, Francisco Román-Dañobeytia, Waldo Lavado-Casimiro

**Affiliations:** 1 Servicio Nacional de Meteorología e Hidrología (SENAMHI), Calle Cahuide 785 - Jesús María, Lima, 11 Peru; 2 Consorcio para el Desarrollo Sostenible de la Ecorregión Andina (CONDESAN), Calle Las Codornices 253 - Surquillo, Lima, 34 Peru; 3 Iniciativa Regional de Monitoreo Hidrológico de Ecosistemas Andinos (iMHEA), Av. Ricardo Palma 698 - Miraflores, Lima, 18 Peru; 4 Department of Civil and Environmental Engineering, Imperial College London, London SW7 2AZ, UK; 5 ATUK Consultoría Estratégica, Luis Pasteur y Copérnico, Cuenca 010105, Ecuador; 6 Forest Trends, 1203 19th Street, N.W., 4R Washington D.C. 20036, USA

**Keywords:** Environmental sciences, Ecology, Climate sciences, Hydrology

## Abstract

A new FAO Penman-Monteith reference evapotranspiration gridded dataset is introduced, called PISCOeo_pm. PISCOeo_pm has been developed for the 1981–2016 period at ~1 km (0.01°) spatial resolution for the entire continental Peruvian territory. The framework for the development of PISCOeo_pm is based on previously generated gridded data of meteorological subvariables such as air temperature (maximum and minimum), sunshine duration, dew point temperature, and wind speed. Different steps, i.e., (i) quality control, (ii) gap-filling, (iii) homogenization, and (iv) spatial interpolation, were applied to the subvariables. Based on the results of an independent validation, on average, PISCOeo_pm exhibits better precision than three existing gridded products (CRU_TS, TerraClimate, and ERA5-Land) because it presents a predictive capacity above the average observed using daily and monthly data and has a higher spatial resolution. Therefore, PISCOeo_pm is useful for better understanding the terrestrial water and energy balances in Peru as well as for its application in fields such as climatology, hydrology, and agronomy, among others.

## Abstract (summary)

**Table:** 

Measurement(s)	reference evapotranspiration • air temperature • sunshine duration • dew point temperature • wind speed
Technology Type(s)	weather station
Factor Type(s)	reference evapotranspiration
Sample Characteristic - Environment	Climatology • Hydrology • Meteorology • Ecology • Earth Science
Sample Characteristic - Location	Peru • Andes

## Background & Summary

Evapotranspiration plays an essential role in terrestrial, water, and to a lesser extent, carbon energy cycles^1–6^. Terrestrial evapotranspiration is the water transferred from the land surface to the atmosphere and is generally parameterized as a sum of soil evaporation, vegetation evaporation, and vegetation transpiration^3^. The evapotranspiration rate of a reference surface (a hypothetical grass reference crop with specific characteristics), which occurs without water restrictions, is known as the reference evapotranspiration (ET_o_)^1^. ET_o_ is a variable of great interest for estimating actual evapotranspiration in agronomy, for example, from the crop coefficients.

ET_o_ can be calculated from meteorological data, and the climatic parameters are the only factors that affect its estimation^7^. The most accepted formula for calculating ET_o_ and the one used in this work is that of FAO Penman-Monteith (FPM)^1^. The main obstacle to apply FPM is the availability of information in meteorological stations, because data on maximum and minimum air temperature, solar radiation (sunshine duration), actual vapour pressure (dew point temperature), and wind speed are needed but usually absent. An interesting study developed in Ecuador^8^, a country with climatic characteristics comparable to Peru, showed that the absence of some of the variables (subvariables) for the calculation of FPM can lead to unreliable estimates. It was found that using estimated wind speed data has no major effect on calculated ET_o_; however, solar radiation data can yield erroneous estimations of ET_o_ by as much as 24%. If relative humidity data are estimated indirectly, the error may be as high as 14%; and if all data except air temperature are estimated indirectly, errors might be larger than 30%. In general, the impact of the lack of information on subvariables in the FPM procedure depends on the climatic condition (humid or arid), yielding different results according to the study region^8–11^. In the case of Peru, air temperature data are readily available, while other subvariables are quite limited^12,13^. The few estimates of ET_o_ in Peru use methods based on air temperature^14–16^. Certain local studies have tried to estimate ET_o_ based on FPM with limited data. Lavado *et al*.^17^ developed an empirical FPM correction equation based on the Hargreaves-Samani formula^18^ for the Peruvian Andean-Amazon region, while Laqui *et al*.^19^ used artificial neural networks (ANNs) for the determination of ET_o_ and suggested techniques based on ANNs to estimate it in the presence of few variables. Although the FPM formula was applied in these studies to determine ET_o_, they used different methods to estimate solar radiation indirectly, a variable that is particularly scarce in Peru. Lavado *et al*.^17^ based their study on the results of Baigorria *et al*.^20^, who estimated coefficients at national scale for determining solar radiation, while Laqui *et al*.^19^ determined solar radiation according to data on sunshine duration in the Department of Puno, southern Peru.

Satellite data, such as surface temperature or cloud cover, are very useful to develop a uniform density of climate information, especially in countries such as Peru where the density of meteorological stations is low and spatially scattered. The availability of gridded ET_o_ data in Peru is limited and almost nonexistent, and there is only one experimental product of evapotranspiration^21^ at the time of development of this research, which is estimated based on the formula of Oudin^22^. This product should be used with caution since it refers to potential evapotranspiration and not ET_o_^23^. Despite the above, it is still possible to directly obtain ET_o_ or variables that allow its estimation from global gridded products. However, these gridded data might not provide all the necessary data or may not reasonably represent the spatiotemporal variability of the variables in areas where few stations have been used for gridding. For example, CRU_TS^24^ and TerraClimate^25^ provide a variety of variables as well as the direct estimation of ET_o_, but their precision in a given area is conditioned by the number of stations (in CRU_TS) or the grid data used (in TerraClimate). On the other hand, the use of global reanalysis products such as NCEP/NCAR^26^, ERA5^27^, and ERA5-Land^28^, among others, can provide the necessary variables for the estimation of ET_o_ at high temporal resolution. However, its coarse spatial resolution can be considered a crucial disadvantage. Therefore, this shortcoming would indicate a need to produce gridded ET_o_ data at the national scale and with a finer spatial resolution.

Considering these limitations, the product PISCOeo_pm is a gridded database at a resolution of 1 km built from a process of preliminary elaboration of gridded meteorological subvariables necessary to apply the FPM formula at a daily rate during 1981–2016. PISCOeo_pm is developed by the National Service for Meteorology and Hydrology of Peru (SENAMHI) and is part of the Peruvian Interpolated data of SENAMHI’s Climatological and Hydrological Observations (PISCO) with gridded data on precipitation^29^, air temperature^13^, and flow rates^30^ at the scale of all of Peru. The PISCOeo_pm product and the gridded data of the subvariables for its estimation are freely available. We argue that the PISCOeo_pm dataset is the best available estimate of ET_o_ using high spatial resolution FPM in Peru, especially under an estimation context of regionally complex topography and limited data. Its application is expected to be useful in different fields, such as climatology, hydrology, and agronomy, among others.

## Methods

### Overview

The main problem in the estimation of ET_o_ is its high data requirement, usually affected by data discontinuity, quality control problems, missing data, and inhomogeneities^31–33^. This is crucial in topographic complex areas with little spatial coverage of meteorological stations such as Peru^12,13,29^. In this sense, there is a severe difficulty to obtain all the required subvariables in one single meteorological station to estimate ET_o_^17^. When certain subvariables are not available, two types of approaches have been used to allow ET_o_ calculations^7,8,23,34^: (i) using methods that require fewer subvariables, commonly known as “less demanding methods”, and (ii) estimating missing data before ET_o_ calculation.

In the “less demanding methods”, the use of approaches requiring only air temperature data, such as Hargreaves-Samani^18^, among others^23^, is still common, mainly because air temperature is commonly available. Nevertheless, one of the major drawbacks of these methods is that variability and trends in the estimated ET_o_ values depend only on air temperature, regardless of the importance of the other subvariables^8,19,35,36^.

In the case of estimating missing data before ET_o_ calculation, two options are also possible: i) use the recommendations given by the FPM document^1^, and ii) use neighbour meteorological stations. However, whenever observed data corresponding to the non-observed subvariables is obtainable from nearby stations, the use of FPM recommendations should be avoided. This is because they use stationary relationships between variables that were empirically derived, which can be problematic in the context of climate change since these relationships may also change^7^. The same problem affects the “less demanding methods”, which rely on empirical relationships^36^.

**Fig. 1:**
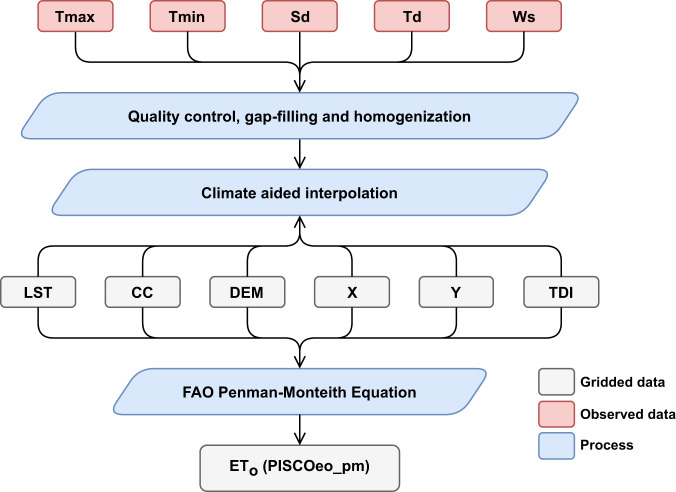
Workflow diagram for the development of PISCOeo_pm. Meteorological subvariables include maximum air temperature (Tmax), minimum air temperature (Tmin), sunshine duration (Sd), dew point temperature (Td), and wind speed (Ws). Spatial covariables include land surface temperature (LST), cloud cover frequency (CC), elevation (DEM), longitude (X), latitude (Y), and topographic dissection index (TDI).

The use of nearby meteorological station data to estimate missing data takes advantage of spatiotemporal interpolation methods. It is the only approach mentioned above that estimates missing data using information about the same variable. This strategy is known as interpolating first, calculating later (IC), and has two main steps. First, the missing variables are estimated using a spatial interpolation method, and second, the FPM is calculated. This method was tested in various world regions^7,37–39^, and there is evidence that this approach yields better results^36^ than the former. Therefore, we used the IC strategy to determine PISCOeo_pm. The flowchart of the IC process (Fig. 1) involves several steps, which include quality control, gap-filling, and homogenization of the subvariables of maximum air temperature (Tmax), minimum air temperature (Tmin), sunshine duration (Sd), dew point temperature (Td), and wind speed (Ws). Then, the climatologically aided interpolation of these subvariables is performed with the support of spatial covariables to finally apply the FPM formula at a gridded level and obtain ET_o_, i.e., PISCOeo_pm.

In this work, the subvariables Sd, Td, and Ws were analysed. The Tmax and Tmin data are drawn from a gridded database called PISCOt^13^, which underwent a process of spatial downscaling to be consistent with the rest of the aforementioned subvariables.

### Observational source data

Observed data from meteorological stations of Sd, Td and Ws across Peru were provided by the SENAMHI. We used information from Sd to determine solar radiation, and Td to determine actual vapour pressure. As mentioned, Tmax and Tmin were not directly used as these had been previously estimated.

**Fig. 2:**
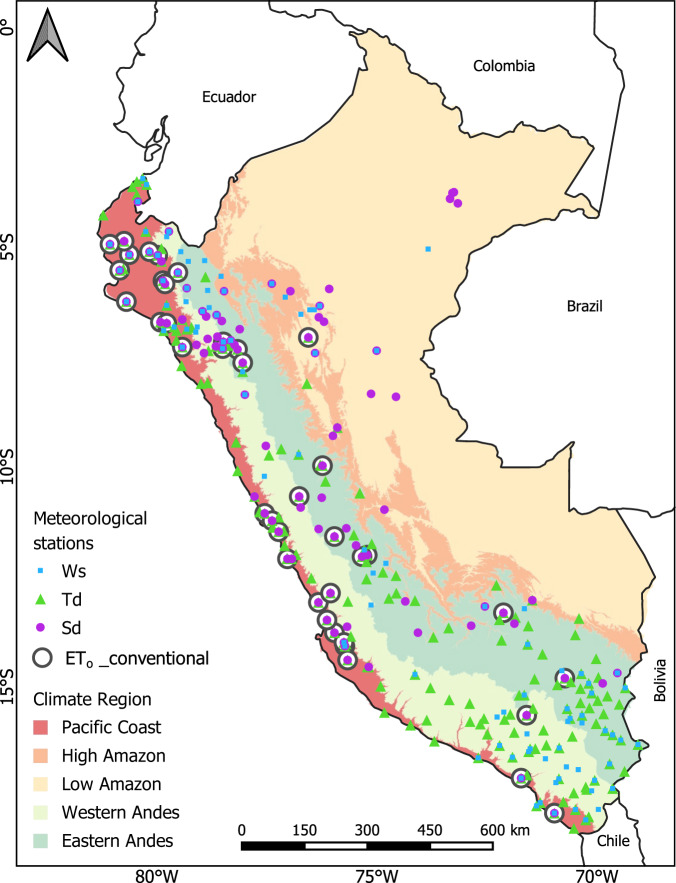
Map of the final set of stations used for the generation of gridded data of meteorological subvariables: sunshine duration (Sd), dew point temperature (Td), and wind speed (Ws). The map includes the chosen meteorological stations used for the PISCOeo_pm framework validation (ET_o__conventional). Boundaries represent the main climate regions of Peru.

**Fig. 3:**
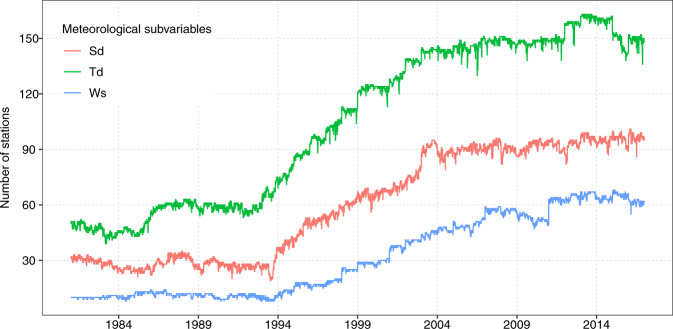
Temporal distribution of meteorological stations for sunshine duration (Sd), dew point temperature (Td), and wind speed (Ws) from 1981 to 2016.

The raw dataset included more than 300 stations from the initial process (Table 1 and Supplementary Fig. 1). The spatial distribution of the stations is adequate, especially in the Andean and coastal regions, but there is a lack of stations in the Peruvian lowland Amazon forest (Fig. 2). Although it is true that the density of meteorological stations is not very large, this number and distribution exceeds those in previous studies, such as those determining solar radiation in Peru^20,40^. Data availability during the period 1981–2016 is shown in the Fig. 3. It is observed that for these three variables, the amount of data available has increased since 1995, whereas a lower data availability is observed during the period 1981–1995 by about 70% less (Fig. 3). This decrease is mainly caused due to the social and political instability in the country at that time^12^. The final number of stations used for the spatial interpolation was 93, 182, and 99 for Sd, Td, and Ws, respectively (Table 1 and Fig. 2).

**Table 1:** Summary of the number of stations, main procedure and spatial predictors for the spatial model (climate normals) for each meteorological subvariable.

Variable	Procedure	N original stations	N level 3 stations	N stations validation	Unit	Spatial predictor
Maximum air temperature (Tmax)	Spatial downscaling	—	—	39	°C	LST_day, DEM
Minimum air temperature (Tmin)		—	—	39	°C	LST_night, DEM
Sunshine duration (Sd)	Spatial interpolation	357	93	39	hours	CC, DEM, X, Y
Dew point temperature (Td)		434	182	39	°C	LST_mean, DEM, X, Y
Wind speed (Ws)		434	99	—	m/s	WS_worldclim

N original stations indicates the original information obtained; N level 3 stations indicate the amount of information after the process of quality control, gap-filling, and homogenization; N stations for validation indicate the stations that were used for the PISCOeo_pm framework validation (ET_o__conventional). Spatial covariables include daytime land surface temperature (LST_day), nighttime land surface temperature (LST_night), mean land surface temperature (LST_mean), cloud cover frequency (CC), elevation (DEM), longitude (X), latitude (Y), and WorldClim wind speed (WS_worldclim).

#### Quality control

Data quality control (QC) was performed for daily values of Sd, Td, and Ws and consisted of five stages:

1. Control of coding errors: where coding errors are identified in sequences of n days with a repeated value. The following records were classified as suspect data: for the Sd, sequences of 15 days with records of 0 sun hours and sequences of 10 days with a repeated value greater than 0; in the Td series, sequences of 7 days with a repeated value; and for the Ws series, sequences of 15 days with a repeated value. These limits are based on the QC methodology of Tomas Burguera *et al*.^7^.
2. Control of out-of-physical-range values: based on physical thresholds of the variables. In Sd, its lower limit was 0, corresponding to a cloudy day, and its upper limit corresponded to the maximum value of sun hours as a function of latitude. In the Td data, the lower limit was −25 °C, and the upper limit was 30°C. In the case of Ws, the lower physical limit was 0 m/s, corresponding to a day without notable winds, and the upper limit was 40 m/s. These limits were based on the suspect record thresholds of Tomas Burguera *et al*.^7^.
3. Control of out-of-climate-range values: the extreme values were evaluated for the three variables as a function of the climatologies of the time series of each analysed station. The algorithm identifies the records that are above the 3rd quartile plus m times the interquartile range (IQR) and those that are below the 1st quartile minus m times the IQR (m: 3.5 for Sd and Td; m: 4.5 for Ws).
4. Comparison with neighbouring stations: a comparison was made of the percentile rank^41^ of the daily series of a target station with the four closest stations, which meet the requirements of being within 70 km and an elevation difference less than 1000 m^42,43^ (Supplementary Fig. 2). The approach of comparing the difference in the percentile series allows the identification of only the extreme records as recommended by Tomas Burguera *et al*.;^7^ the established thresholds were 0.8 for Sd and Td and 1 for Ws. After the first four QC steps were completed, the records that had been identified in at least one stage of the QC processes were eliminated.
5. Visual control: we proceeded with a visual inspection^32,33^ of the time series of each station to identify annual periods with inhomogeneities that could not be corrected. To do so, plots of daily time series and yearly frequencies of decimal numbers were inspected, and the periods with inconsistent records were eliminated.

#### Gap-filling

The simple interpolation of incomplete data can produce artificial inhomogeneities in the final gridded product because of the changing spatial coverage of the stations during the period 1981–2016^29,44–46^. This might impact on the variance and lead to erroneous conclusions about data changes and variability^47^. To avoid such a situation, a gap-filling procedure was performed prior to spatial interpolation.

To complete missing information, a gap-filling algorithm was used based on neighbouring stations using standardized data^48^. The purpose of performing standardization was to avoid differences in the mean and variance. The standardization was performed through the daily climatology of the subvariables.

Two conditions were required to consider a station as neighbour: i) five years of data in common (365 days repeated at least five times) and a minimum correlation of 0.6^45,49–51^. Likewise, to make use of those stations that do not share a common period at the beginning, an iterative application of the algorithm^52^ of at least three cycles was developed to search for neighbouring stations according to the horizontal-vertical distance (Supplementary Fig. 2) of (i) 70 km-1000 m, (ii) 100 km-1000 m, and (iii) 150 km-(no vertical limit).

To obtain a greater number of complete time series during the analysis period (1981–2016), virtual stations obtained from ERA5-Land from the equivalent subvariables were used. These series were corrected using empirical quantile mapping^53,54^ based on anomalies (preserving the daily climatology). This process was used only for the time series with seven years of data. The virtual stations that had a minimum correlation of 0.55 (with the target station after correction) were preserved to be used as a neighbouring station for the gap-filling procedure.

Originally, the gap-filling algorithm was applied to air temperature time series. Certain specifications were made for its application in Sd: the lower limit is always 0, and the upper limit can be as high as the observed data reached (between 10 and 13 hours).

#### Homogenization

The homogenization of the time series after the data gap-filling process was performed by applying the standard normal homogeneity test (SNHT)^55^ in its relative and absolute version. The relative version is known as the pairwise homogenization algorithm (PHA), originally developed by Menne and Williams^56^. Relative homogenization was performed as long as a target station had at least eight neighbouring stations (with a correlation equal to or greater than 0.6). The search for neighbouring stations was established at a distance and elevation difference of 1000 km and 1000 m, respectively (Supplementary Fig. 2). If the above was not possible, absolute homogenization was considered, that is, the single application of the test to the target station. Similar to gap-filling, homogenization was performed in three cycles.

The homogenization of the time series was carried out on a monthly scale; therefore, the correction was at that same scale. To carry out this correction at the daily scale, monthly factors were interpolated on a daily basis^57^. The number of series observed after gap-filling and homogenization for each subvariable is indicated in Table 1.

### Spatial predictors

For the grid generation of the meteorological subvariables (Sd, Td, and Ws), the support of spatial covariables obtained from satellite products were used. Table 2 presents the datasets and related predictors used in the spatial regression models (Table 1).

**Table 2:** Gridded databases and related covariables to be used in the spatial interpolation of meteorological subvariables.

Database	Variable	Abbreviation	Spatial Resolution	Temporal Resolution
MOD11A2	Land surface temperature day, night and mean	LST_day, LST_night, LST_mean	Global, 1 km	Daily 2000–2019
EarthEnv	Cloud cover frequency	CC	Global, 1 km	Climatology 2000–2014
GMTED2010	Elevation	DEM	Global, 1 km	—
WorldClim 2.1	Wind speed	WS_worldclim	Global, 1 km	Climatology 1979–2000

Land surface temperature (LST) values were obtained from the Moderate Resolution Imaging Spectroradiometer (MODIS)^58^, 8-day, 1 km product (MOD11A2)^59^. Monthly LST means for both day (LST_day) and night (LST_night) observations (2000–2019), as its average value (LST_mean), were used. For a single 8-day grid value, if the MODIS quality assurance flags indicate cloud contamination or other possible issues resulting in an average emissivity error >0.02 or average LST error >2 °C, we did not consider the value in the 20-year mean. Missing data in the final grid was reconstructed through a nearest-neighbour interpolation. LST data were downloaded from https://developers.google.com/earth-engine/datasets/catalog/MODIS_006_MOD11A2.

Cloud cover (CC) frequency data is derived from 15 years climatology (2000–2014) developed by Wilson and Jetz^60^, which is based on twice-daily observations making use of product MOD09GA and MYD09GA cloud flags at a 1 km resolution. Data were downloaded from http://www.earthenv.org/cloud.

The elevation (DEM) variable was extracted from the 1 km Global Multi-resolution Terrain Elevation Data 2010 (GMTED2010)^61^. Longitude (X), latitude (Y) and the topographic dissection index (TDI) were derived at the same spatial resolution from DEM. The TDI was calculated through a multiscale calculation of the DEM. The TDI value for a specific window size represents the height of a grid cell relative to the surrounding terrain^62^. Here, the multiscale TDI was calculated for a total of five spatial window sizes (3, 6, 9, 12, and 15 km)^45^. DEM data were downloaded from https://developers.google.com/earth-engine/datasets/catalog/USGS_GMTED2010.

Additionally, we used the wind speed Worldclim (WS_worldclim) dataset (WorldClim 2.0)^63^. WorldClim is a global gridded product (1 km) of monthly average data (1970–2000), based on spatial interpolation using thin-plate splines of a high number of meteorological station observations, with covariates including elevation, distance to the coast and other satellite data. Data were downloaded from http://www.worldclim.com/version2.

Finally, the data were re-gridded to the same spatial extension and at 0.01°, which corresponds to the final resolution of PISCOeo_pm.

### Spatial interpolation

The spatial interpolation approach used is called climatologically aided interpolation^64–66^, where the average (climate normal) values are prioritized independently of their daily values. This approach greatly reduces computational costs (compared to applying it independently by time) and preserves the average variability of ET_o_ at the national scale. There is evidence that this approach is more efficient than performing the process by time step independently, at least for air temperature^66^. In addition, the covariables may not necessarily need to be in the same temporal range as the observed data. As such, the interpolation is divided into three steps:

1. Spatial interpolation of normal values of the meteorological subvariables.
2. Spatial interpolation of anomalies with respect to normal values.
3. Aggregation of 1. and 2. to obtain the actual value of the meteorological subvariables.

**Fig. 4:**
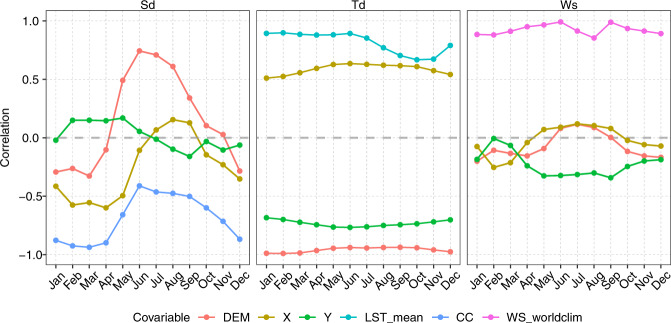
Spatial correlation of sunshine duration (Sd), dew point temperature (Td), and wind speed (Ws) with spatial covariables at the normal monthly scale (average).

For steps 1. and 2., the regression-kriging method^67,68^ is used, where both normals and anomalies can be expressed as the sum of the deterministic and stochastic components. In step 1., a different combination of spatial covariables is used for each meteorological subvariable (Table 1). This step is based on the high spatial correlation among the data (Fig. 4). Covariable selection is very important in this study because of the very low availability of meteorological data. In step 2., the same covariables as step 1. are used plus the TDI.

It should be mentioned that Sd and Td were interpolated in both normals (1981–2010) and anomalies (1981–2016). Ws was only interpolated in normals, due to the lack and poor quality of observed data. For Ws, the average values correspond to the available data (with more than 5 years of data) during 1981–2016 (Table 1). Some studies have shown that the absence of Ws has a minimal impact on the estimation of ET_o_^8^ and that using an average value that changes in space is much better than using a constant value^69^.

### Spatial downscaling of Tmax and Tmin

It is important to note that previously, Huerta *et al*.^13^ described the construction of the gridded data of Tmax and Tmin at the scale of Peru (PISCOt). These gridded data were at a resolution of ~10 km (0.1°), and to be used in the construction of PISCOeo_pm, the spatial scale was reduced using two covariables: LST and DEM (Tables 1 and 2). Reducing the spatial scale of PISCOt was inspired by other works in which the geographically weighted regression (GWR)^70,71^ technique was applied^72–74^. The methodology used is divided into three steps:

1. Spatial downscaling considering the normals values (1981–2010).
2. Spatial downscaling of anomalies with respect to normal values (1981–2016).
3. Aggregation of 1. and 2. to obtain the actual values of Tmax and Tmin.

In step 1., GWR was applied at a resolution of 0.1° for both PISCOt and for LST and DEM. The coefficients (and residuals) of the obtained model were interpolated using the bilinear approach (BL) at 0.01°. Then, these values were used to estimate Tmax and Tmin as a function of LST (LST_day and LST_night) and DEM (using a spatial resolution of 0.01°). For step 2., BL was used to directly downscale the anomalies from 0.1° to 0.01°. In step 3., both sets of data at a resolution of 0.01° were simply added. The approach used can be considered a climatologically aided spatial downscaling since the reduction is prioritized in only the average (normal) values independently of their daily values^64,65^.

### Generation of ET_o_ gridded data

To determine PISCOeo_pm, the FPM^1^ formula was applied:

1 $$E{T}_{o}=\frac{0.408\Delta ({R}_{n}-G)+\gamma \frac{900}{T+273}{u}_{2}({e}_{s}-{e}_{a})}{\Delta +\gamma (1+0.34{u}_{2})}$$

where ET_o_ is the reference evapotranspiration (mm day^−1^), *R*_*n*_ is the net radiation at the crop surface (MJ m^−2^ day^−1^), *G* is the soil heat flux density (MJ m^−2^ day^−1^), *T* is the average daily air temperature at 2 m height (°C), *u*_2_ is the wind speed at 2 m height (m s^−1^), *e*_*s*_ is the saturation vapour pressure (kPa), *e*_*a*_ is the actual vapour pressure (kPa), Δ is the slope of the vapour pressure curve (kPa °C^−1^), and *γ* is the psychrometric constant (kPa °C^−1^).

In this context, since the approach to generating a gridded ET_o_ is the IC type, the gridded data of the subvariables were previously determined. The subvariables in the FPM formula were applied as follows:

- *T* was obtained from the average value between Tmax and Tmin.
- *R*_*n*_ was determined as the difference between the incoming net shortwave radiation and the outgoing net longwave radiation:

2 $${R}_{n}={R}_{ns}-{R}_{nl}$$

where *R*_*ns*_ is the net solar or shortwave radiation (MJ m^−2^ day^−1^), and *R*_*nl*_ is the net outgoing longwave radiation (MJ m^−2^ day^−1^).

*R*_*ns*_ results from the balance between incoming and reflected solar radiation and is given by:

3 $${R}_{ns}=(1-a){R}_{s}$$

where *a* is the albedo or canopy reflection coefficient, which is 0.23 for the hypothetical grass reference crop (dimensionless); and, *R*_*s*_ is the measured or calculated solar radiation (MJ m^−2^ day^−1^).

*R*_*nl*_ is expressed quantitatively by the Stefan-Boltzmann law. The net energy flux leaving the earth’s surface is, however, less than that emitted and given by the Stefan-Boltzmann law due to the absorption and downward radiation from the sky. Water vapour, clouds, carbon dioxide and dust are absorbers and emitters of longwave radiation. Their concentrations should be known when assessing the net outgoing flux. As humidity and cloudiness play an important role, the Stefan-Boltzmann law is corrected by these two factors when estimating the net outgoing flux of longwave radiation. It is thereby assumed that the concentrations of the other absorbers are constant:

4 $${R}_{nl}=\sigma \left[\frac{{({\rm{T}}{\rm{m}}{\rm{a}}{\rm{x}}+273.16)}^{4}+{({\rm{T}}{\rm{m}}{\rm{i}}{\rm{n}}+273.16)}^{4}}{2}\right]\left(0.34-0.14\sqrt{{e}_{a}}\right)\left[1.35\frac{{R}_{s}}{{R}_{so}}-0.35\right]$$

where *σ* is the Stefan-Boltzmann constant (4.903 10^−9^ MJ K^−4^ m^−2^ day^−1^), and *R*_*so*_ the clear-sky radiation (MJ m^−2^ day^−1^).

Both *R*_*ns*_ and *R*_*nl*_ required *R*_*s*_ as key variable. *R*_*s*_ was calculated by applying the Angstrom formula^1^, which relates with Sd:

5 $${R}_{s}=\left({a}_{s}+{b}_{s}\times \frac{{\rm{S}}{\rm{d}}}{N}\times {R}_{a}\right)$$

where *R*_*a*_ is the extraterrestrial radiation (MJ m^−2^ day^−1^), *N* is the maximum possible duration of sunshine or daylight hours (hours), Sd*/N* is the relative sunshine duration, *a*_*s*_ is the regression constant, expressing the fraction of extraterrestrial radiation reaching the earth on overcast days (Sd = 0), and *a*_*s*_ + *b*_*s*_ is the fraction of extraterrestrial radiation reaching the earth on clear days (Sd = *N*). As no actual solar radiation data were available and no calibration has been carried out for improved *a*_*s*_ and *b*_*s*_ parameters, the values *a*_*s*_ = 0.25 and *b*_*s*_ = 0.50 were used for the study area^1,8,75^.

- *e*_*a*_ is usually estimated as a function of relative humidity. We estimate *e*_*a*_ based on Td, ensuring that *e*_*a*_ is within the physical limits of relative humidity. This was done as follows:

6 $${e}_{a}=0.618\times {e}^{\left[\frac{17.27\times {\rm{T}}{\rm{d}}}{{\rm{T}}{\rm{d}}+237.3}\right]}$$

7 $$rh=({e}_{a}/{e}_{s})100$$

8 $${e}_{a}=\frac{(rh\times {e}_{s})}{100}$$

where *rh* is the relative humidity (%). First, *e*_*a*_ is preliminarily calculated using Td; later, *rh* is obtained as a result of *e*_*a*_ and *e*_*s*_ (based on Tmax and Tmin); next, *rh* is set to maintain a maximum value of 100%; finally, *e*_*a*_ is calculated once again but using the corrected *rh*.

- For the *u*_2_ information, we directly used the gridded normals of Ws as a quasi-constant value; that is, we apply only the values of monthly climatologies for the days that correspond to the given month.
- The latitude and elevation data were obtained directly from the spatial covariables. These data are important because they are input data to determine the astronomical and atmospheric pressure variables.

It should be mentioned that on a daily scale, the value of *G* is relatively low^1^, so it is ignored (*G* = 0) in the calculations. For the design of the ET_o_ formula, this research was based on that described by Zotarelli *et al*.^76^, which follows the guidelines of Allen *et al*.^1^.

## Data Records

The set of gridded data generated consists of geolocated gridded files of the meteorological subvariables (Tmax, Tmin, Sd, Td, and Ws) and of ET_o_ (PISCOeo_pm).

The dataset corresponds to the normal (average) and daily values of Tmax, Tmin, Sd, Td, and Ws, and ET_o_. Each of these is stored in NetCDF format in one file per variable, each defined by three dimensions (*time*, *latitude*, and *longitude* represented by date, latitude, and longitude, respectively). Only in the normal files, the time dimension refers to the numerical value of a month of the year (starting from January). For practical reasons, the gridded data were divided into different repositories and are collected in figshare^77^

## Technical Validation

The construction of PISCOeo_pm has been evaluated in four parts: i) gap-filling validation of Sd and Td; ii) independent validation of ET_o_; iii) comparison with existing global products of ET_o_; and, iv) estimate of the uncertainty of ET_o_.

The metrics used to characterize the accuracy, precision and goodness of fit of the data were simple error (bias), mean absolute error (MAE), and the refined index of agreement (*d*_*r*_)^78,79^. The *d*_*r*_ metric varies between −1 and 1, with a value of >0.5 indicating a predictive power greater than the observed average. The *d*_*r*_ is similar to a correlation coefficient, but a high value indicates both high correlation and low absolute differences between the observed and model time series^80^.

The global ET_o_ products used in this section were CRU_TS^24^, TerraClimate^25^, and ERA5-Land^28^. The ET_o_ data in CRU_TS are at a spatial resolution of 0.5° and on a monthly scale from 1901 to the present. Because in CRU_TS, the monthly value of ET_o_ represents the average (and not the cumulative), this value was multiplied by the number of days to obtain a representation of the cumulative value of ET_o_. TerraClimate has a spatial resolution of ~4 km, and ET_o_ is at a cumulative monthly scale (1958–2020). It should be mentioned that ET_o_ in TerraClimate was not estimated from FPM but, instead, using the formula of the American Society of Civil Engineers (ASCE). The FPM and ASCE formulas are quite similar, and the difference lies in the constant values that refer to whether the reference crop is short or tall. ERA5-Land is a reanalysis product that contains a great diversity of surface variables at a spatial resolution of 9 km (~0.1°) since 1981. In ERA5-Land, there is no variable approximate to ET_o_; therefore, this was estimated on a daily scale using the subvariables in Eq. 1.

### Gap-filling validation of Sd and Td

A gap-filling procedure was done for extending shorter meteorological stations (back to 1981) before the construction of PISCOeo_pm. To evaluate the infilled values accuracy, we calculate the statistical metrics (bias, MAE and *d*_*r*_) by comparing measured and estimated data for available dates with observed values. This was only performed for the Sd and Td time series (Ws was not gap-filled due to the lack and poor quality of observed data).

**Fig. 5:**
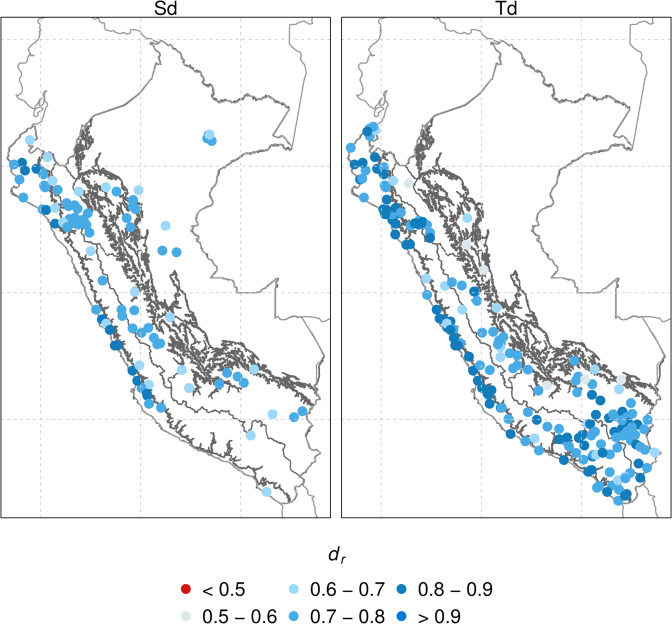
Spatial distribution of *d*_*r*_ for the gap-filling validation in sunshine duration (Sd) and dew point temperature (Td).

Table 3 summarizes the statistical metrics for both subvariables, and Fig. 5 shows the spatial distribution of *d*_*r*_. The infilling models exhibited that Sd and Td are well reproduced, with a median *d*_*r*_ of 0.76 and 0.78, respectively. There is a slightly better performance of Td than Sd. In addition, we can observe that most stations present a *d*_*r*_ > 0.65 for all climate regions. High (low) efficiency tends to be present in areas with a high (low) density of meteorological stations. It should be mentioned that we did not find *d*_*r*_ < 0.5 in any station, demonstrating that the used approach worked well.

**Table 3:** Gap-filling error statistics for daily (1981–2016) sunshine duration (Sd) and dew point temperature (Td).

Variable	bias	MAE	*d*_*r*_
Sunshine duration (Sd)	0.05	1.25	0.76
Dew point temperature (Td)	0.01	0.95	0.78

Overall, the validation errors for the infilling models appeared to be satisfactory well, especially considering the complex topographic area and the lack of meteorological stations. The use of virtual stations (from ERA5-Land) and the preservation of the daily climatology may explain the positive results.

### Independent validation of PISCOeo_pm

The PISCOeo_pm framework validation follows the “Train/Test Split” technique, dividing station data into training and test data. The test data were 39 meteorological stations that met the criteria of presenting all the sub-variables (Tmax, Tmin, Sd, Td, and Ws) and are called ET_o__conventional (Fig. 2 and Table 1). The Tmax and Tmin series went through similar processing to Td, and Ws was obtained from the interpolated product (at the grid point of the station location). We calculated ET_o_ in ET_o__conventional by applying the same procedure of the FPM formula. The training data was the original data but omitted ET_o__conventional.

The PISCOeo_pm framework is executed again but uses the training data to obtain a gridded product, called PISCOeo_pm_for_cv. Once this stage is completed, we tested ET_o__conventional with PISCOeo_pm_for_cv using the statistical metrics of bias, MAE and *d*_*r*_ to evaluate the PISCOeo_pm framework. Additionally, the gridded global products are assessed. Therefore, ET_o__conventional is compared with PISCOeo_pm_for_cv, CRU_TS, TerraClimate, and ERA5-Land.

**Fig. 6:**
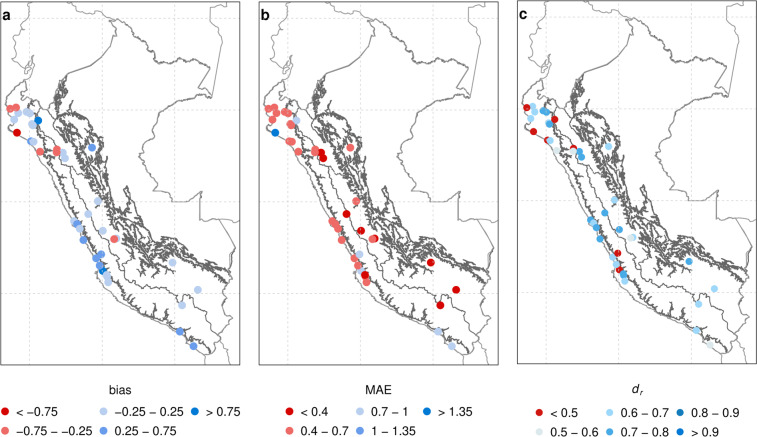
Spatial distribution of statistical metrics (bias (**a**); MAE (**b**); and *d*_*r*_ (**c**)) of ET_o__conventional versus PISCOeo_pm_for_cv at daily scale.

The Fig. 6 shows the bias, MAE, and *d*_*r*_ metrics for ET_o__conventional versus PISCOeo_pm_for_cv at the daily scale (1981–2016). It is observed that the statistical metrics are acceptable for the meteorological stations located (mainly) in the Andes but show a reduced efficiency in some stations of the coastal area (PC). In addition, these statistics show less efficiency for monthly (Supplementary Fig. 3) and annual (Supplementary Fig. 4) accumulations; PISCOeo_pm_for_cv, in general, has a low performance according to the *d*_*r*_ metric, *d*_*r*_ < 0.5 in all stations.

The comparisons of ET_o__conventional with the three global products at different aggregation frequencies (daily, monthly, annual, and normal monthly), including the PISCOeo_pm_for_cv data, are shown as a summary in Table 4. These results indicate that PISCOeo_pm_for_cv exhibits a higher performance (*d*_*r*_ > 0.5) at all aggregation scales and tends to present the lowest bias and highest accuracy (bias and MAE) concerning the other products. However, only at annual temporal resolution, the three statistical metrics show low efficiency. None of the products has an *d*_*r*_ greater than 0.5, and only PISCOeo_pm_for_cv is close to 0. For this purpose, the best gridded products according to the independent validation (in hierarchical order) were PISCOeo_pm_for_cv, ERA5-Land, TerraClimate, and CRU_TS. The low values of the metrics at the annual scale are due to the accumulation of the daily biases of ET_o_ at the annual scale.

**Table 4:** Summary (median) of statistical metrics (bias, MAE, and *d*_*r*_) for ET_o__conventional versus PISCOeo_pm_for_cv, CRU_TS, TerraClimate, and ERA5-Land at different levels of aggregation.

Product	Temporal Resolution	bias (mm)	MAE (mm)	*d*_*r*_
PISCOeo_pm_for_cv	daily	0.08	0.52	0.67
ERA5-Land		−0.22	0.69	0.55
PISCOeo_pm_for_cv	monthly	2.39	11.7	0.66
CRU_TS		−14.3	16.18	0.37
TerraClimate		−16.51	16.75	0.35
ERA5-Land		−6.7	16.18	0.54
PISCOeo_pm_for_cv	yearly	28.67	80.46	−0.06
CRU_TS		−171.55	182.75	−0.53
TerraClimate		−198.18	198.18	−0.51
ERA5-Land		−80.42	162.13	−0.45
PISCOeo_pm_for_cv	normal monthly	2.39	10.74	0.66
CRU_TS		−14.3	15.09	0.33
TerraClimate		−16.51	16.51	0.31
ERA5-Land		−6.7	15.33	0.54

Overall, the evaluation demonstrates that PISCOeo_pm_for_cv reproduces reasonably well ET_o_ (ET_o__conventional). This is critical as PISCOeo_pm_for_cv corresponds to a worst-case scenario of fewer available meteorological stations compared to PISCOeo_pm. It must be made clear that we do not intend to disfavor the use of the global products but merely to highlight how they differ. These datasets are useful for specific purposes and can be improved in future versions.

### Comparison of ET_o_ in PISCOeo_pm with global products

The ET_o_ values obtained from PISCOeo_pm (1981–2016) were compared at daily, monthly, and annual scale with three global products (CRU_TS, TerraClimate, and ERA5-Land) and for five climate regions (Fig. 2): Pacific Coast (PC), High Amazon (HA), Low Amazon (LA), Western Andes (WA), and Eastern Andes (EA).

**Fig. 7:**
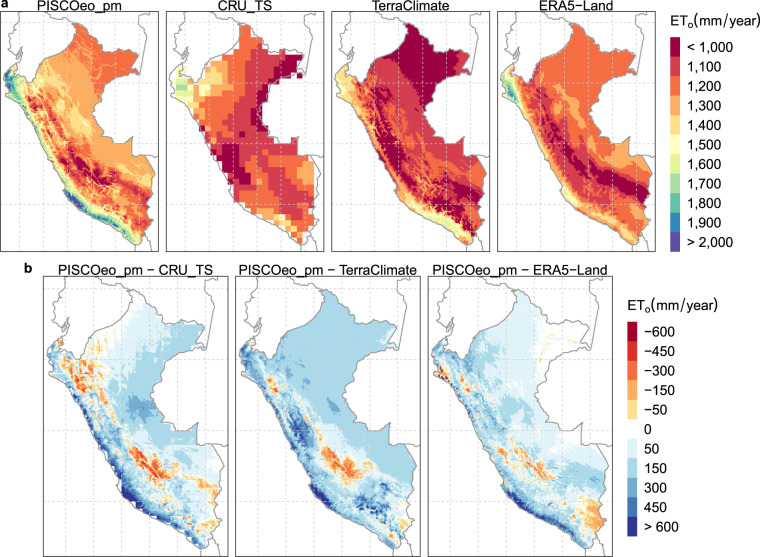
Spatial distribution and differences of the mean annual (1981–2010) reference evapotranspiration (ET_o_). (**a**) Spatial distribution for PISCOeo_pm, CRU_TS, TerraClimate, and ERA5-Land. (**b**) Difference of PISCOeo_pm with each global product.

Figure 7a illustrates the spatial distribution of the ET_o_ annual average (1981–2010) for PISCOeo_pm, CRU_TS, TerraClimate, and ERA5-Land. It is shown that ERA5-Land presents the best spatial similarity compared to PISCOeo_pm, especially in PC. Considering the differences in the magnitude of PISCOeo_pm with the three products (Fig. 7b), it was found that PC presents the most significant magnitude differences, reaching values of more than 600 mm. In the Amazon area (HA and LA), ERA5-Land and CRU_TS are closer to PISCOeo_pm (between 0–150 mm) than TerraClimate (between 150–300 mm). It is important to note that the Andes area (EA and WA) exhibits the most complex spatial variability (positive and negative differences), highlighting only ERA5-Land that shows the smallest magnitudes.

**Fig. 8:**
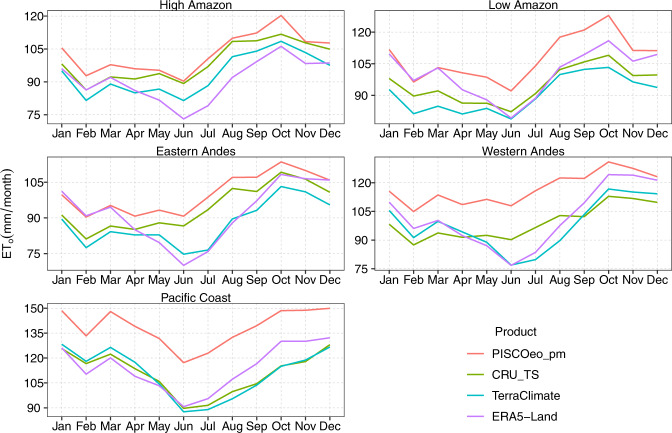
Monthly climatology (1981–2010) of reference evapotranspiration (ET_o_) for PISCOeo_pm, CRU_TS, TerraClimate, and ERA5-Land in the different climatic regions.

For comparison purposes, monthly climatologies and annual values (1981–2016) were calculated in the global products and PISCOeo_pm at the regional climate scale. The monthly variability in each of the five regions is observed in Fig. 8, where it is evident that the three products tend to underestimate the values with respect to PISCOeo_pm, being quite marked in PC and WA for all months; however, CRU_TS follows the signal of the monthly pattern better than the other products.

**Fig. 9:**
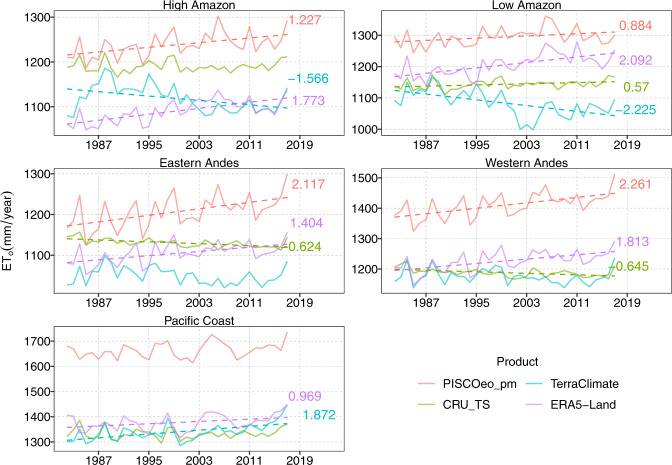
Annual variability (1981–2016) in reference evapotranspiration (ET_o_) for PISCOeo_pm, CRU_TS, TerraClimate, and ERA5-Land in the different climatic regions. When the slope was significantly different from 0, the slope value was shown on the right of the plots.

The annual variability (1981–2016) of ET_o_ for PISCOeo_pm, CRU_TS, TerraClimate, and ERA5-Land at each climate region is illustrated in Fig. 9. Additionally, the Sen’s slope is shown (rate of change during 1981–2016) whether it presents a significant trend (*p* value < 0.05). As observed in the monthly climatologies, ET_o_ is notably underestimated by the three global products, being in PC quite considerable. PISCOeo_pm shows significant positive trends in all regions except in the PC, but its signal tends to increase. Under this premise of upward trends in the five regions, TerraClimate shows different trend directions compared to those of PISCOeo_pm in all regions except PC. Regarding the products that are closest to the PISCOeo_pm slopes (magnitude and direction), ERA5-Land stands out in PC, EA, WA, HA, and CRU_TS in LA.

Further analysis using the statistical metrics (Table 5) revealed that ET_o_ in PC and WA regions show the most remarkable deviations. On a daily and monthly scale, ERA5-Land presents notable similarities in the LA and EA regions with PISCOeo_pm. For monthly aggregations, CRU_TS shows a comparable signal in the HA and EA regions, but TerraClimate does not excel in any area, showing low values of the statistical metrics. At the annual time scale (Supplementary Table 1), the three products evaluated indicate high dissimilarities with PISCOeo_pm since their *d*_*r*_ metrics are negative in all regions. At the normal monthly level (Supplementary Table 1), the results are similar to those found on daily and monthly scales.

**Table 5:** Statistical metrics (bias, MAE, and *d*_*r*_) of PISCOeo_pm versus CRU_TS, TerraClimate, and ERA5-Land for the different climatic regions and aggregation levels (daily and monthly) during 1981–2016.

Climatic Region	Product	Temporal Resolution	bias (mm)	MAE (mm)	*d*_*r*_
PC	ERA5-Land	daily	−0.61	0.62	0.25
HA			−0.4	0.42	0.45
LA			−0.24	0.35	0.65
WA			−0.5	0.53	0.19
EA			−0.28	0.35	0.51
PC	CRU_TS	monthly	−27.66	27.66	−0.31
HA			−4.01	4.76	0.71
LA			−12.6	12.63	0.33
WA			−18.48	18.48	−0.13
EA			−6.38	6.96	0.58
PC	TerraClimate		−27.05	27.07	−0.29
HA			−10.01	10.15	0.38
LA			−17.58	17.61	0.07
WA			−19.53	19.54	−0.18
EA			−13.1	13.14	0.2
PC	ERA5-Land		−18.66	18.66	0.03
HA			−12.32	12.32	0.25
LA			−7.42	8.02	0.58
WA			−15.29	15.4	0.04
EA			−8.51	9.27	0.43

The results presented show an inherent disparity of ET_o_ between the gridded products. PISCOeo_pm seems to have a seasonal and overall distribution similar to the global products (mainly ERA5-Land) but presents a greater magnitude. Generally, we see that the differences tend to increase from east (Amazon) to west (Pacific), i.e., from humid to arid regions, respectively^81^. Singer *et al*.^82^ also notes this behaviour at a global scale, but using different databases with other evapotranspiration formulations. As in this analysis, we focused on global products based on the FPM formulation; the same conclusion of Singer *et al*.^82^ can not be reached. There are, however, other possible explanations. The discrepancy between PISCOeo_pm and the global products could be attributed to the low spatial resolution of ET_o_ and the inherent bias in the meteorological subvariables. For instance, the coarse spatial resolution of CRU_TS can not represent complex terrain features of Peru, leading to substantial differences. Although TerraClimate and ERA5-Land provide a better spatial resolution, biases in solar radiation could be presented. This is crucial because solar radiation errors have the highest impact on the estimated ET_o_^8,9,19^. A recent study proved that satellite-based solar radiation estimates are more accurate than ERA5-Land solar radiation outputs, implying a more accurate ET_o_ by using the satellite-based estimation^83^. In addition, it could be mentioned that depending on the season, some term (aerodynamic or radiation) of FPM exerts a significant role in the estimation of ET_o_, resulting in that other subvariables can be as influential as solar radiation. Further research needs to closely examine the subvariables and their bias effect in ET_o_ calculation.

Finally, it should be mentioned that PISCOeo_pm is a product that presents a homogenization procedure prior to gridding the data, a task that few products globally perform because it is rather complicated. This process is demonstrated quite well in the trend analysis, where there is divergence (TerraClimate, for example, was not designed for this type of analysis^25^) in the direction of the trends in different climatic regions; only ERA5-Land tends to preserve these characteristics. This evaluation demonstrates that PISCOeo_pm can be useful for understanding the historical variability of ET_o_ as other global products.

### Uncertainty estimation of ET_o_

We estimated the uncertainty of ET_o_ or PISCOeo_pm (Δ ET_o_) using the error propagation approach, which is designed to quantify the effect of variables uncertainty on the error of a function that combined these variables, in order to provide an accurate estimate of function error^84–86^. The error caused by each variable on the estimation of ET_o_ can be approximated by finite differences of each variable (in the FPM formula) and adding them to estimate Δ ET_o_. Studies using this approach are often based on sensitivity analysis (of each variable in the FPM formula) and only use data from meteorological stations^87,88^. In this work, we carried out the error propagation approach at the grid scale, therefore, Δ ET_o_ was obtained for each day during 1981–2016.

**Fig. 10:**
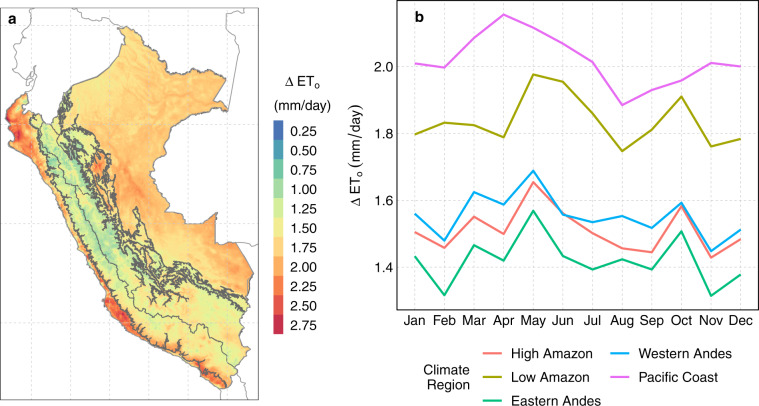
Uncertainty of reference evapotranspiration (Δ ET_o_) during 1981–2016: spatial annual average (**a**), and monthly climatology (**b**) variability.

For an easier interpretation of the uncertainty of ET_o_, annual and monthly averages were calculated from the daily Δ ET_o_. Figure 10a shows the annual average and monthly climatology of Δ ET_o_ during 1981–2016. At annual scale, the highest Δ ET_o_ were found in the coastal region (north and center-south), reaching values of up to 2.75 mm/day. A similar behaviour, but to a lesser extent, was also found in the Amazon region where Δ ET_o_ reaches 1.5–2.25 mm/day. Only the north and center areas of the Andes had lower values of Δ ET_o_, ranging between 0–1 mm/day. At monthly scale (Fig. 10b), Δ ET_o_ had a weak seasonality (with slightly higher Δ ET_o_ values during March to August) and confirmed that the greatest (minor) errors were found in the climatic regions PC and LA (EA).

It should be mentioned that Δ ET_o_ is a measure of uncertainty associated with the FPM formula (of the input variables) and does not include other sources of uncertainty such as those derived from the input variable preparation process (quality control, gap-filling, homogenization, spatial interpolation, and spatial downscaling). Due to the methodological framework of PISCOeo_pm, an estimation of the total uncertainty is a complicated task. Despite the above, we believe that the estimation of Δ ET_o_ is useful for the different applications of PISCOeo_pm since it characterises areas of greater and lesser uncertainty of ET_o_.

## Usage Notes

Although the main variable is ET_o_ (that is, PISCOeo_pm), we also provide data for the meteorological subvariables. The technical validation only prioritized verifying ET_o_ but not Sd, Td, and Ws, so the use of these variables alone should be determined by the user. The intention of sharing these data is based on the fact that they can be used for other applications in conjunction with other variables already established in Peru, such as precipitation and air temperature.

Additionally, due to the high spatial resolution of the gridded data, it is possible to obtain estimates of the subvariables and ET_o_ in water bodies (lakes and rivers). However, these grids should be considered empty grids (masked) due to the very nature of ET_o_ and to the use of spatial covariables (for example, greater validation is required to confirm the accuracy of the spatial patterns of Tmax, Tmin, and Td over water due to the use of LST). Therefore, gridded data should only be used in continental areas.

According to the results, PISCOeo_pm is able to characterize the temporal trends of ET_o_. This make PISCOeo_pm a useful dataset to understanding the historical variability of ET_o_. However, we recommend that any trend analysis should also use meteorological stations as much as possible.

The FPM formulation is the most accurate formula so far and has been widely used within the last two decades. However, considering a distinct vegetation response to elevated CO_2_, it is important to point out that some of the assumptions that underlie the computation of FPM are incorrect under conditions of changing CO_2_ concentrations. A basic assumption in FPM is that the minimum surface resistance over a wet surface is fixed and is thus explicitly assumed to show no response to changing CO_2_. This assumption is ultimately not valid for vegetated surfaces as the minimum stomatal resistance is expected to increase with increasing CO_2_^89–98^. Although this takes more importance for future evaluations, it is also presumed to be valid for the recent decade. In this study, we did not apply the aforementioned modification; therefore, a bias on the ET_o_ values could be produced in the last years. This is expected to be taken into account in future versions of PISCOeo_pm.

Finally, it should be mentioned that PISCOeo_pm will be updated approximately every two years, which is a similar period to that of the meteorological subvariables. In addition, we expect to incorporate new observations (especially from neighbouring countries and for the Amazon area) that improve the overall quality of the dataset.

## Supplementary information

SUPPLEMENTARY INFORMATION

## Data Availability

Construction of the gridded data was performed using the R environment for statistical computing version 3.6.3. Python version 3.8.5 was also used. The code that describes the procedures (quality control, gap-filling, homogenization, spatial interpolation, and spatial downscaling) to obtain the gridded data of the meteorological subvariables and PISCOeo_pm is freely available at figshare^99^ and GitHub (https://github.com/adrHuerta/PISCOeo_pm) under GNU public licence version 3.
