# Supplementary material for: PISCOeo_pm, a reference evapotranspiration gridded database based on FAO Penman-Monteith in Peru
Source: Sci Data. 2022 Jun 17;9:328. doi: 10.1038/s41597-022-01373-8 (PMC9205949; doi:10.1038/s41597-022-01373-8)
Supplement: Supplementary file 1 — SUPPLEMENTARY INFORMATION [file 41597_2022_1373_MOESM1_ESM.pdf]

Supplementary Information for "PISCOeo_pm, a reference evapotranspiration gridded database based on FAO Penman-Monteith in Peru"

Adrian Huerta[1,*], Vivien Bonnesoeur[2,3], José Cuadros-Adriazola[2,3,4], Leonardo Gutierrez[1], Boris F. Ochoa-Tocachi[3,4,5,6], Francisco Román-Dañobeytia[2,3], and Waldo Lavado-Casimiro[1]

[1]Servicio Nacional de Meteorología e Hidrología (SENAMHI), Calle Cahuide 785 - Jesús María, Lima 11, Perú.
[2]Consorcio para el Desarrollo Sostenible de la Ecorregión Andina (CONDESAN), Calle Las Codornices 253 - Surquillo, Lima 34, Perú.
[3]Iniciativa Regional de Monitoreo Hidrológico de Ecosistemas Andinos (iMHEA), Av. Ricardo Palma 698 - Miraflores, Lima 18, Perú.
[4]Department of Civil and Environmental Engineering, Imperial College London, London SW7 2AZ, UK
[5]ATUK Consultoría Estratégica, Luis Pasteur y Copérnico, Cuenca 010105, Ecuador.
[6]Forest Trends, 1203 19th Street, N.W., 4R, Washington D.C. 20036, USA.
[*]corresponding author(s): Adrian Huerta (adrhuerta@gmail.com)

Supplementary Figures

- Supplementary Figure 1. Map of the initial set of stations used for the generation of gridded data of meteorological subvariables: sunshine duration (Sd), dew point temperature (Td), and wind speed (Ws). Boundaries represent the main climate regions of Peru.
- Supplementary Figure 2. Analysis of the number of available stations (a) and correlation (b) versus distance versus elevation for each meteorological subvariables: sunshine duration (Sd), dew point temperature (Td), and wind speed (Ws).
- Supplementary Figure 3. Spatial distribution of statistical metrics (bias (a); MAE (b); and d_r (c)) of ET_o_conventional versus PISCOeo_pm_for_cv at monthly scale.
- Supplementary Figure 4. Spatial distribution of statistical metrics (bias (a); MAE (b); and d_r (c)) of ET_o_conventional versus PISCOeo_pm_for_cv at yearly scale.

Supplementary Tables

- Supplementary Table 1. Statistical metrics (bias, MAE, and d_r) of PISCOeo_pm versus CRU_TS, TerraClimate, and ERA5-Land for the different climatic regions and aggregation levels (yearly and normal monthly) during 1981–2016.

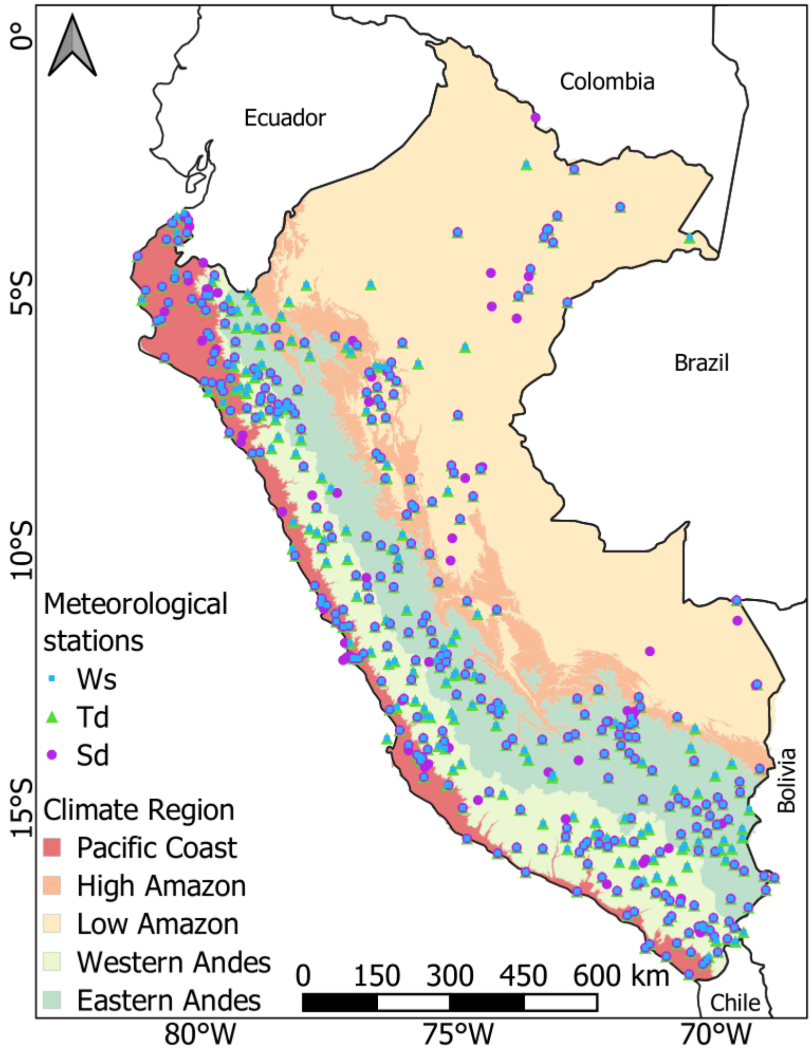

Supplementary Figure 1. Map of the initial set of stations used for the generation of gridded data of meteorological subvariables: sunshine duration (Sd), dew point temperature (Td), and wind speed (Ws). Boundaries represent the main climate regions of Peru.

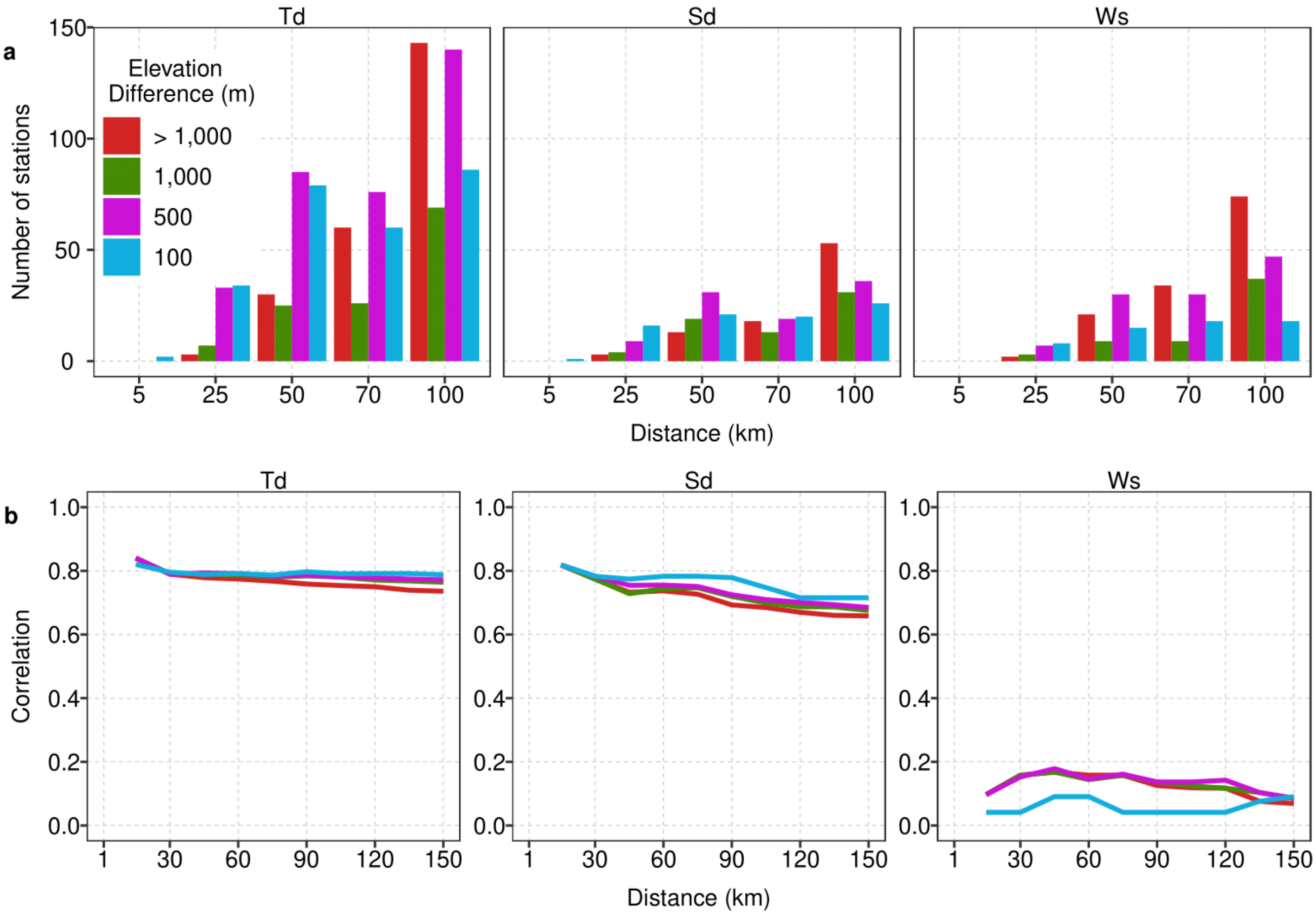

Supplementary Figure 2. Analysis of the number of available stations (a) and correlation (b) versus distance versus elevation for each meteorological subvariables: sunshine duration (Sd), dew point temperature (Td), and wind speed (Ws).

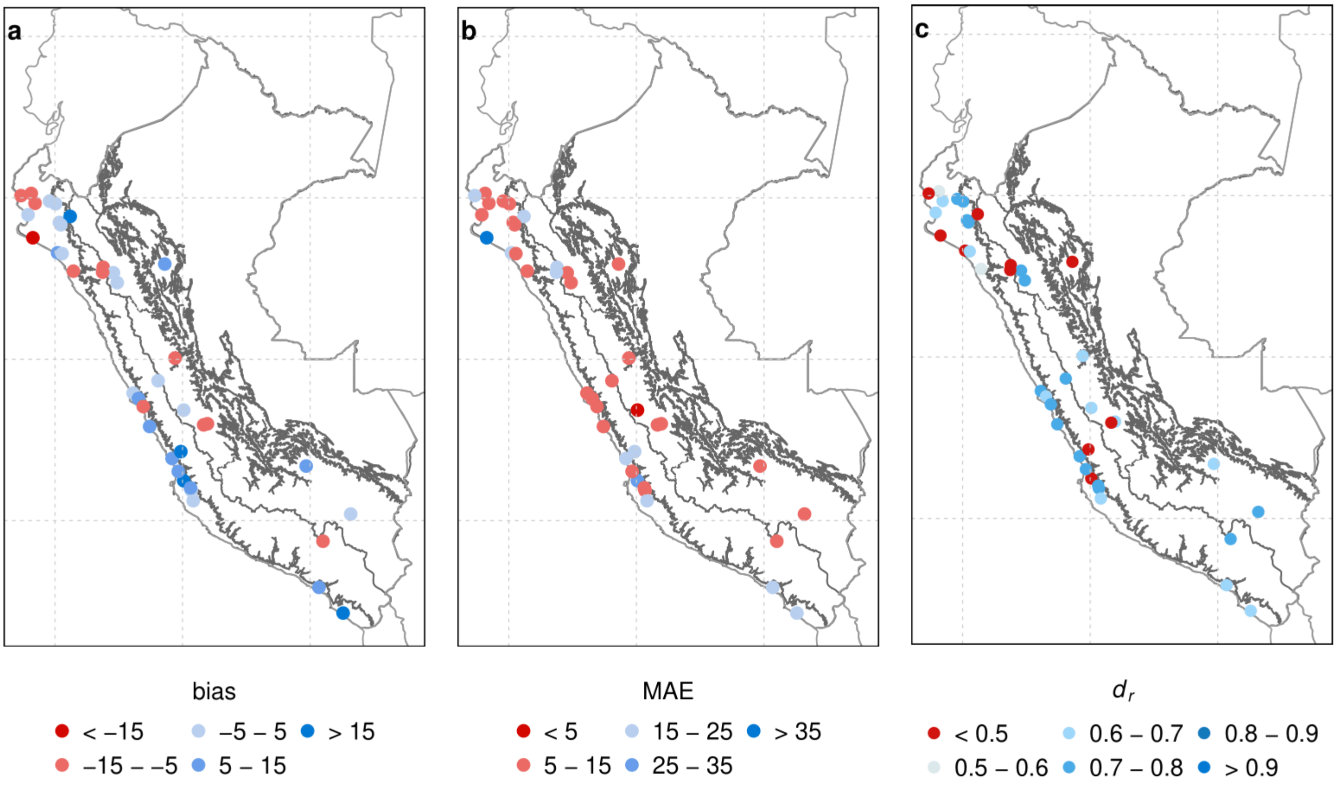

Supplementary Figure 3. Spatial distribution of statistical metrics (bias (a); MAE (b); and d_r (c)) of ET_o_conventional versus PISCOeo_pm_for_cv at monthly scale.

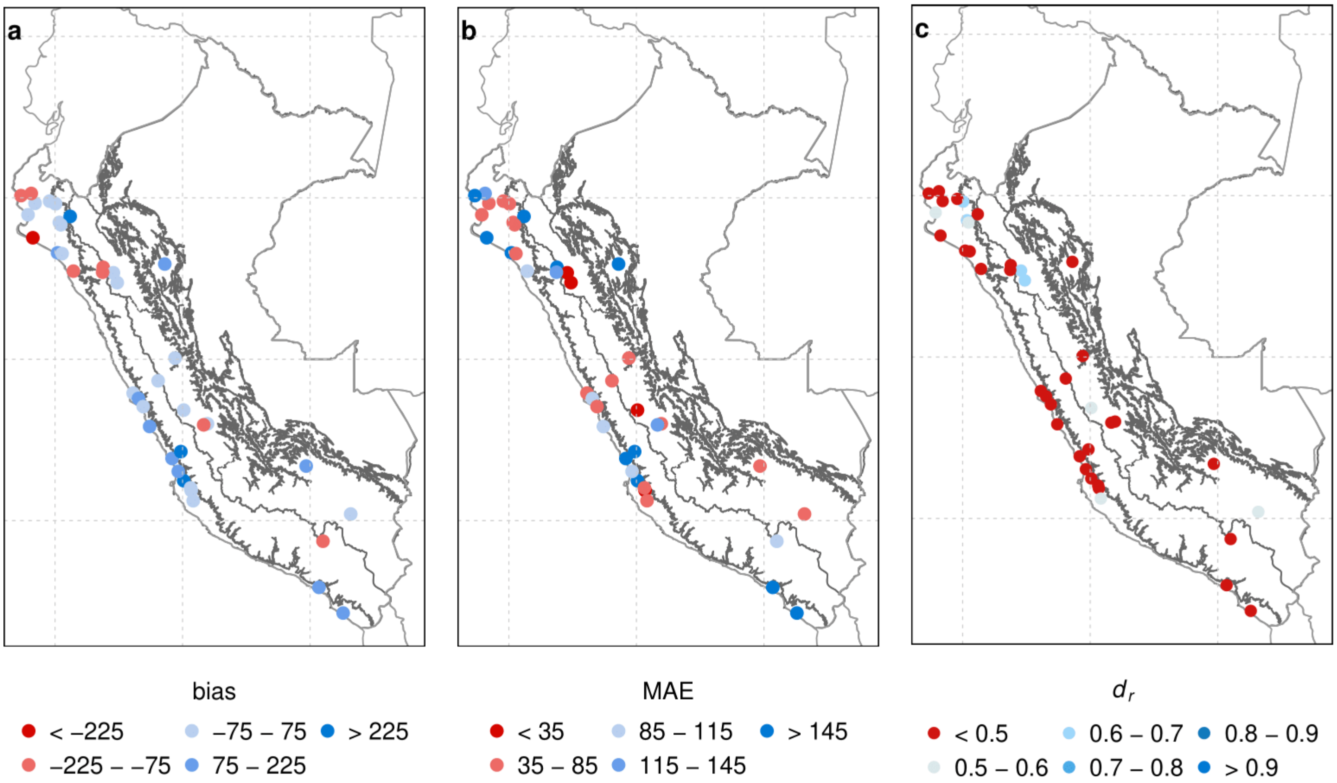

Supplementary Figure 4. Spatial distribution of statistical metrics (bias (a); MAE (b); and d_r (c)) of ET_o_conventional versus PISCOeo_pm_for_cv at yearly scale.

Climatic Region	Product	Temporal Resolution	bias (mm)	MAE (mm)	d_r
PC	CRU_TS	yearly	-331.88	331.88	-0.85
HA			-48.06	48.06	-0.13
LA			-151.17	151.17	-0.73
WA			-221.73	221.73	-0.71
EA			-76.57	76.57	-0.16
PC	TerraClimate		-324.61	324.61	-0.85
HA			-120.08	120.08	-0.65
LA			-211.02	211.02	-0.81
WA			-234.37	234.37	-0.73
EA			-157.26	157.26	-0.59
PC	ERA5-Land		-223.86	223.86	-0.78
HA			-147.89	147.89	-0.72
LA			-89	89	-0.54
WA			-183.52	183.52	-0.65
EA			-102.15	102.15	-0.37
PC	CRU_TS	normal monthly	-27.66	27.66	-0.35
HA			-4.01	04.01	0.74
LA			-12.6	12.6	0.3
WA			-18.48	18.48	-0.25
EA			-6.38	6.38	0.56
PC	TerraClimate		-27.05	27.05	-0.34
HA			-10.01	10.01	0.35
LA			-17.58	17.58	0.02
WA			-19.53	19.53	-0.29
EA			-13.1	13.1	0.09
PC	ERA5-Land		-18.66	18.66	-0.04
HA			-12.32	12.32	0.2
LA			-7.42	7.53	0.58
WA			-15.29	15.29	-0.09
EA			-8.51	8.75	0.39

Supplementary Table 1. Statistical metrics (bias, MAE, and d_r) of PISCOeo_pm versus CRU_TS, TerraClimate, and ERA5-Land for the different climatic regions and aggregation levels (yearly and normal monthly) during 1981–2016.
